# Estimating the population health impact of a multi-cancer early detection genomic blood test to complement existing screening in the US and UK

**DOI:** 10.1038/s41416-021-01498-4

**Published:** 2021-08-23

**Authors:** Allan Hackshaw, Sarah S. Cohen, Heidi Reichert, Anuraag R. Kansal, Karen C. Chung, Joshua J. Ofman

**Affiliations:** 1 Cancer Research UK & University College London Cancer Trials Centre, London, UK; 2 EpidStrategies, A Division of ToxStrategies, Inc, Cary, NC USA; 3 EpidStrategies, A Division of ToxStrategies, Inc, Ann Arbor, MI USA; 4 GRAIL, Inc., 1525 O’Brien Drive, Menlo Park, CA USA

**Keywords:** Epidemiology, Oncology

## Abstract

**Background:**

Multi-cancer early detection (MCED) next-generation-sequencing blood tests represent a potential paradigm shift in screening.

**Methods:**

We estimated the impact of screening in the US and UK. We used country-specific parameters for uptake, and test-specific sensitivity and false-positive rates for current screening: breast, colorectal, cervical and lung (US only) cancers. For the MCED test, we used cancer-specific sensitivities by stage. Outcomes included the true-positive:false-positive (TP:FP) ratio; and the cost of diagnostic investigations among screen positives, per cancer detected (Diag_cost_). Outcomes were estimated for recommended screening only, and then when giving the MCED test to anyone without cancer detected by current screening plus similarly aged adults ineligible for recommended screening.

**Results:**

In the US, current screening detects an estimated 189,498 breast, cervical, colorectal and lung cancers. An MCED test with 25–100% uptake detects an additional 105,526–422,105 cancers (multiple types). The estimated TP:FP (Diag_cost_) was 1.43 ($89,042) with current screening but only 1:1.8 ($7060) using an MCED test. For the UK the corresponding estimates were 1:18 (£10,452) for current screening, and 1:1.6 (£2175) using an MCED test.

**Conclusions:**

Adding an MCED blood test to recommended screening can potentially be an efficient strategy. Ongoing randomised studies are required for full efficacy and cost-effectiveness evaluations.

## Background

There are an estimated 18.1 million newly diagnosed cases of cancer worldwide and 9.6 million cancer deaths (2018) [1]. New cancer cases in the US are expected to exceed 1.8 million in 2020 [2] with 46% detected at regional or distant stage disease [3]. In the UK, 367,000 cancer diagnoses were made annually from 2015 to 2017; 45% at regional or distant stage at diagnosis [4]. The clinical costs of cancer care are significant with $201 billion (2019 dollars) estimated to be spent in the US during 2020 [5] and £21 billion in the UK in 2018 [6].

Screening aims to detect cancer at an earlier stage for which there are effective, potentially curative treatments (colorectal and cervical screenings aim both to detect and treat pre-invasive lesions) [7, 8]. There are currently only four recommended population-level screening programmes: breast, lung, colorectal, and cervical cancers, because of a favourable benefit-harm balance [9] Together, these four cancers represent only 29% of total cancer incidence and 24% of cancer-related deaths in the US among individuals aged 50–79 (Supplemental Fig. 1). Also, adherence is below national targets in the US and England [10, 11]. In the US, prostate screening is only recommended on an individualised basis. There is, as yet, no effective screening test for all other cancer types, and many are unlikely to ever be associated with cost-effective single-cancer screening programmes because they each have relatively low incidence and mortality.

Cancer screening is currently based on the principle of one test for one cancer type. Recent high-profile publications of genomic technologies (using next-generation sequencing) describe blood tests that can detect signals from multiple cancers, some with impressive preliminary screening performance: examples are Galleri, CancerSEEK and PanSeer [12–14]. A multi-cancer early detection (MCED) test using a single blood draw has obvious appeal [15]. Additionally, diagnostic tests (scans and biopsies) are expensive [16, 17], and an MCED test with a very low false-positive rate (FPR) could be a highly cost-effective approach.

Our study aimed to produce national estimates of screening performance measures and financial costs of diagnostic investigations for current screening alone (their combined impact), and then when an MCED blood test is employed in the US and UK, which have fundamentally different healthcare systems. This provides public health policy makers and healthcare professionals involved in screening with the first ever examination of how future MCED blood tests might substantially improve screening efficiency. This information can also incentivise research groups to further develop and refine their own tests.

## Methods

The potential impact of 1 year of screening within an ongoing screening programme including an MCED blood test when used alongside current screening was modelled in the US and UK, focusing on clinically detected incident cancers. The target populations were (for 2020) 107,000,000 adults aged 50–79 (US), and 21,834,470 adults aged 45–74 (UK). The UK age range was different because published incidence figures are given in groups of 45–54, 55–64 and 65-74. Country-specific incidence for each cancer type were used [3, 18]. An outline of the modelling is given below with fuller details in Supplemental Text 1.

### Current guideline-recommended screening

USPSTF guidelines recommend screening for breast (mammography), cervical (cytology and high-risk human papillomavirus) and colorectal (multiple tests in use including faecal DNA test and colonoscopy) cancer based on age alone, and lung cancer screening using low-dose computed tomography for adults aged 55–80 who have ≥ 30 pack-year smoking history and currently smoke or have quit within the past 15 years [9, 19–22]. In the US, multiple screening tests are available for colorectal screening, and we use the combination faecal immunochemical test (FIT)-DNA test (FIT-DNA) as the base case, having intermediate sensitivity and specificity compared to other tests. The UK breast and cervix cancer screening recommendations are the same as in the US [23], and FIT testing is often used for colorectal screening. Published national estimates of screening uptake (proportion of eligible individuals in the population who are screened) in both countries were used [11, 24], and also screening performance (sensitivity and FPR) for each test [19–24] [sensitivity: proportion of people with cancer who have a positive test; FPR: proportion of people without cancer who have a positive test, but for cervical screening a false positive is when a woman has colposcopy but no underlying CIN 2 or 3 or invasive cancer]. In general, test performance was assumed to be equal in both the US and UK, but the reported false-positive rate for mammography for the UK was taken from the UK Breast Screening Programme (2.8%) [25]. The estimates of uptake of breast and lung cancer screening we used for the US tended to be higher than reported in some studies, and we assumed all women had annual mammography (as indicated by the American Cancer Society for some age groups, while USPSTF recommend biennial screening). This was done to increase the number of cancers found by current screening, which reduces the number available to be detected by MCED testing (thus making outcomes and costs less favourable for MCED testing). The number of cancers detected by current guideline-recommended screening was computed by multiplying the number of cancers covered by each screening type, the adherence to that screening guideline, and the sensitivity of the screening modality. This approach assumes any interval cancers among cancers with a current guideline-recommended screen are only due to gaps in adherence or the sensitivity of the screening modality.

### MCED test

Screening performance of an MCED blood test was based on an earlier version of the Galleri test, which utilises targeted methylation analysis of circulating cell-free DNA (cfDNA) to detect multiple cancer types [14]. Published sensitivities for each cancer type and stage were used in the modelling (Supplemental Table 2; for interest, the overall sensitivity is 55%), and FPR 0.7% [14]. Incremental cancers detected by the MCED test for each cancer type are computed by multiplying the cancers that are not detected by current guideline-recommended screening, sensitivity of the MCED test for that cancer type, and adherence to MCED testing. Cancers not detected by current screening guidelines include all cancers without a screening test, and only interval cancers for those with a recommended screening test.

### Outcomes

Outcomes per year were (1) total number of individuals with a positive test; (2) total number of individuals diagnosed with cancer following a positive test; (3) true-positive:false-positive (TP:FP) ratio; (4) diagnostic yield, the number of cancers detected as a proportion of the total number screened; (5) cancer detection rate (CDR), the number of cancers detected divided by the total number of cancers expected in the population; (6) total cost of diagnosing detected cancers based on the clinical investigations following a positive test and (7) costs of diagnosing one cancer case detected by screening. The TP:FP ratio is estimated at the point of the screening test result, which would be lower (less favourable) than when calculated based on referral for biopsy. All cancers detected by a screening test were counted. In the case of single-cancer screening only the individual cancers screened for are counted, while for an MCED test the full range of cancers detectable are counted.

### Scenarios

**Fig. 1:**
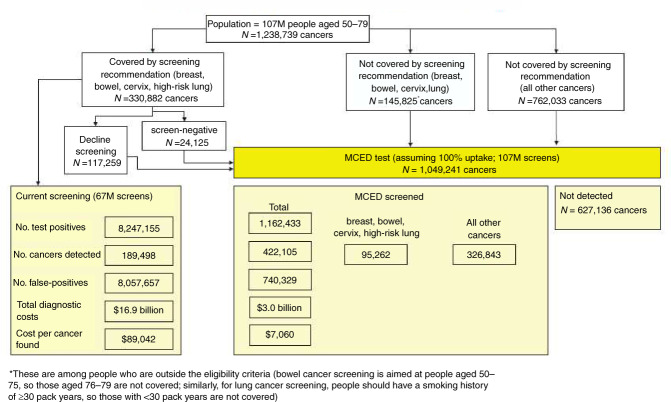
Overview of screening outcomes. Detection pathway and outcomes for cancers found by current recommended standard of care (SOC) screening and the MCED test in our modelling, illustrated for the US among people aged 50–79 years.

Outcomes were estimated for two scenarios outlined in Fig. 1 for the US. The first scenario assumes that individuals are screened using recommended tests only (standard of care), within their eligibility criteria. The second scenario applies an MCED blood test to anyone not diagnosed with breast, bowel, cervical or lung cancer following a positive screen from the currently recommended tests, and also to all adults in the target age group who would be ineligible for recommended screening. We label this the “incremental MCED test.” For the second scenario we initially assumed 100% uptake of the MCED test to reflect the extremes of maximum gains and maximum diagnostic costs. The analyses produced the number of additional cancers (all cancer types) that could be detected by an MCED test during a 1-year period, separate to those found through recommended screening.

### Diagnostic costs following a positive screening test result

After any positive screening test, diagnostic investigations (imaging and biopsies) are required to differentiate true cancer cases from false positives. Diagnostic methods for each cancer type and their unit costs for the US were obtained from the National Comprehensive Cancer Network guidelines and Medicare converted to commercial costs using a 2.3 multiplier [26–28]. For the UK, NICE guidelines [29] and NHS reference tariffs for 2020 [30] were used. Unit costs are in Supplemental Table 3. Costs considered in this study are only those up to the point of diagnosis of a specific cancer type and do not include costs after that point, such as staging costs or treatment costs. Costs for true positives and false positives for the same suspected cancer type are assumed to be equal up to the point of a definitive diagnosis. In addition, we focused on the costs directly associated with the diagnostic workups and have not included costs that would be associated with a screening programme more broadly. For cervical cancer screening (the main purpose is to prevent invasive cancer), so our cost per cancer detected is actually the cost per colposcopy after diagnosis with CIN 2–3 or invasive cancer. Also, diagnostic workups for cervical (colposcopy) and colorectal cancer (removal of polyps during colonoscopy) can prevent future cancer development. Because we only provide outcomes for a single year of screening, no discounting of costs was included. There is also the possibility of having a positive MCED test but diagnostic imaging and other investigations cannot find a tumour. This may be a genuine false positive or a cancer is detected after longer follow-up. There would, therefore, be additional costs of surveillance of these patients, which we have not included.

### Sensitivity analyses

Several sensitivity analyses were conducted. The true-positive calculation assumes that the incidence of cancer among individuals who agree to be screened is the same as in the general population, but it can sometimes be lower (healthier individuals). Therefore, in one sensitivity analysis the incidence of cancers undergoing current screening was decreased by 20%. In a second set of sensitivity analyses we (i) assumed bowel cancer screening in the US is done using colonoscopy, (ii) used the revised (expanded) USPSTF eligibility criteria for lung cancer screening in the US and (iii) added prostate cancer to the current screening paradigm because although there is insufficient evidence in favour of population PSA testing it can be offered to individuals. The third sensitivity analysis assumed 25 and 50% uptake of the MCED blood test instead of 100%. Finally, a subset of the eligible population declines recommended screening (around 20% or 30% for US and UK, respectively); thus, the fourth sensitivity analysis assumed that these individuals would have an MCED blood test.

### Role of the funding source

All authors had full access to all the data in the study and accept responsibility to submit for publication. Three authors are employed by the funding source though this was a collaborative academic study.

## Results

### United States

**Fig. 2:**
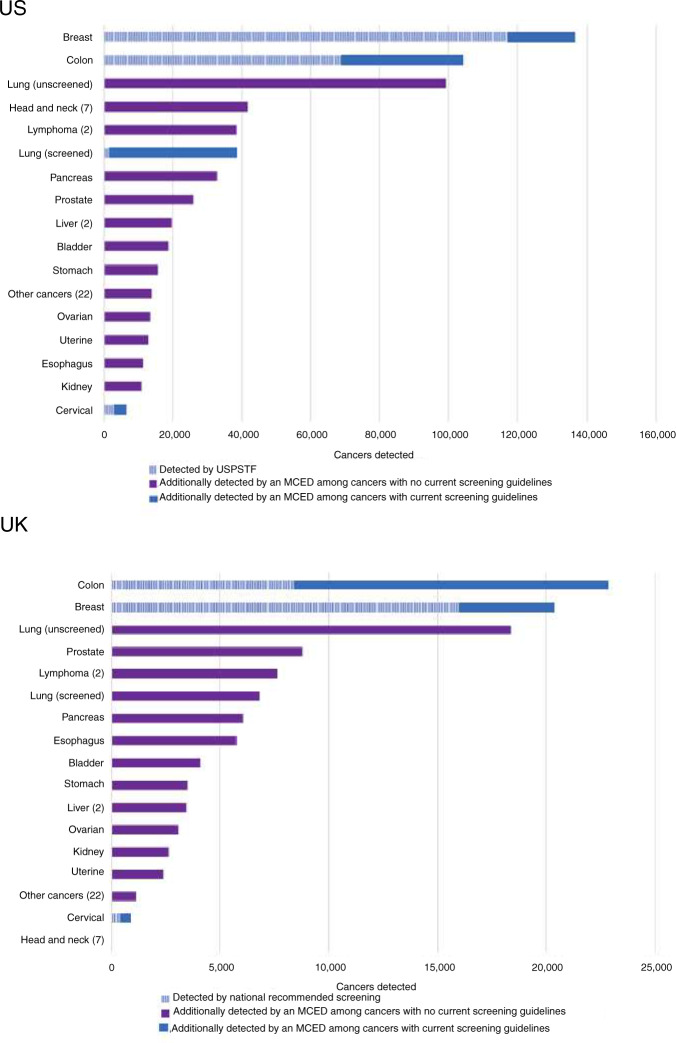
Estimated numbers of cancer detected (in 2020) under current screening paradigms and the additional numbers of cancers detected with an MCED test when used alongside current screening: United States (upper) and United Kingdom (lower). There are seven cancer subtypes grouped under head and neck, two under lymphoma, two under liver and 22 under ‘other’. In total in the US, 189,498 cancers are expected to be detected by USPSTF recommended screening tests and 422,105 additional cancers could be detected by an MCED test. In the UK, 24,888 cancers are expected to be detected by national recommended screening tests and 92,817 additional cancers could be detected by an MCED test.

In the US, an estimated 189,498 breast, lung, colorectal and cervical cancers are found through current recommended screening, with 8,057,657 false positives (Figs. 1 and 2a, Table 1). The TP:FP ratio is therefore 1:43 (to detect one person with any of these four cancers, 43 people without these cancers may have diagnostic investigations following a screen-positive result). The estimate of 189,498 represents 15% of all cancers (CDR). Using an MCED blood test in addition to current recommendations could detect an extra 422,105 cancers (Fig. 2a, Table 1), including 95,262 breast, cervical, colorectal and lung cancers, and 326,843 other cancers, such as head and neck, liver, bladder, stomach, ovary, oesophagus and lymphoma and lung cancer in low-risk individuals (Fig. 2a). This is associated with an additional 1,162,433 test positives of which 740,329 are false positives. Although this represents many additional individuals who may be referred for cancer investigations, the screening efficiency is very high (TP:FP, 1:1.8): to detect one person with cancer only 1.8 people without cancer may undergo diagnostic investigations, among those with a positive MCED test. Furthermore, the CDR is 34%, with an associated 48% increase in diagnostic yield (3.95 vs. 2.66 cancers detected per 1000 screened using MCED testing vs. current screening alone).

**Table 1:** Estimates of screening outcome measures for the US, per year.

		Current screening only					Incremental MCED test				
Analysis	Variation applied for sensitivity analysis	Total positives	Cancers: true positives^a^ (diagnostic yield per 1000^b^)	TP:FP ratio^e^	Cancer detection rate (CDR)^f^	Diagnostic cost per confirmed cancer diagnosis	Total positives	Cancers: true positives^c^ (diagnostic yield per 1000^b^)	TP:FP ratio	Cancer detection rate (CDR)^f^	Diagnostic cost per confirmed cancer diagnosis
Primary Analysis (100% MCED uptake)	–	8,247,155	189,498 (2.66)	1:43	15%	$89,042	1,162,433	422,105 (3.95)	1:1.8	34%	$7060
Sensitivity analyses:	–	–	–	–	–	–	–	–	–	–	–
Decreased cancer incidence in screening population	–20%	8,209,255	151,598 (2.13)	1:53	12%	$110,725	1,179,198	438,870 (4.11)	1:1.7	35%	$6807
Difference*	–	–37,900	–37,900	–	–	$21,683	16,765	16,765	–	–	–$253
Colorectal screening with colonoscopy	Colonoscopy specificity = 100%	5,249,001	189,498 (2.32)	1:27	15%	$77,498	1,162,433	422,105 (3.95)	1:1.8	34%	$7060
Difference*	–	−2,998,154	0	–	–	−$11,544	0	0	–	–	0
Updated USPSTF lung cancer screening	22% of population eligible; 43% of lung cancers	8,544,660	191,652 (2.63)	1:44	15%	$93,328	1,160,868	420,540 (3.95)	1:1.8	34%	$7068
Difference*	–	297,505	2154	–	–	$4286	−1565	−1565	–	–	$8
Prostate cancer added	–	9,001,684	203,810 (2.32)	1:43	16%	$99,589	1,160,815	420,487 (3.94)	1:1.8	34%	$7056
Difference*	–	754,529	14,312	–	–	$10,547	–1618	–1618	–	–	–$4
MCED uptake	50%	–	–	–	–	–	581,217	211,053 (3.95)	1:1.8	17%	$7060
Difference*	–	0	0	–	–	$0	–581,216	–211,053	–	–	$0
MCED uptake	25%	–	–	–	–	–	290,608	105,526 (3.95)	1:1.8	9%	$7060
Difference*	–	0	0	–	–	$0	–871,825	–316,579	–	–	$0
Decline SOC screening^d^	–	N/A	N/A	–	–	N/A	249,252	101,186 (4.74)	1:1.5	8%	$5957
	–	N/A	N/A	–	–	N/A	–913,181	–320,919	–	–	–$1,103

*Difference between sensitivity analysis and primary analysis values.

^a^Only breast, cervix, colorectal and lung cancers.

^b^Yield = cancers detected/total population of screened individuals, expressed per 1000 people.

^c^Any cancer type (including breast, cervix, colorectal and lung cancers missed by the established screening tests).

^d^Assumes 20% of population refuses current screening and only receives an MCED test.

^e^Calculated as (e.g.): 189,498:8247,155–189,498.

^f^CDR = cancers detected /cancers expected = true positives /1,238,739 (from Fig. 1).

The total estimated diagnostic investigation cost associated with current screening was $16.9 billion ($0.4 billion for true positives plus $16.5 billion for false positives), and $3.09 billion for the incremental MCED test assuming the extreme of 100% uptake (Fig. 1), and that in both cases all screen positives undergo further investigations. Although this represents an extra cost, more than double the number of cancers could be detected (422,105 vs. 189,498). The diagnostic cost per cancer detected using the four single-cancer tests is $89,042, but only $7060 with the incremental MCED test.

### United Kingdom

In the UK, an estimated 24,888 breast, colorectal and cervical cancers could be detected with current screening, with 456,988 false positives, representing a TP:FP of 1:18 and a CDR of 12% (Fig. 2b, Table 2). The incremental effect of adding an MCED blood test to the current recommendations could detect an additional 92,817 cancers, with a very low TP:FP of 1:1.6, and the CDR is 43% (Fig. 2b, Table 2).

**Table 2:** Estimates of screening outcome measures for the UK, per year.

		Current screening only					Incremental MCED test				
Analysis	Variation applied for sensitivity analysis	Total positives	Cancers: true positives^a^ (diagnostic yield per 1000^b^)	TP:FP ratio^e^	Cancer detection rate (CDR)^f^	Diagnostic cost per confirmed cancer diagnosis	Total positives	Cancers: true positives^c^ (diagnostic yield per 1000^b^)	TP:FP ratio	Cancer detection rate (CDR)^f^	Diagnostic cost per confirmed cancer diagnosis
Primary Analysis (100% MCED uptake)	–	481,876	24,888 (3.43)	1:18	12%	£10,452	244,153	92,817 (4.26)	1:1.6	43%	£2175
Sensitivity analyses:	–	–	–	–	–	–	–	–	–	–	–
Decreased cancer incidence in screening population	–20%	476,899	19,910 (2.74)	1:23	9%	£12,921	246,220	94,884 (4.35)	1:1.6	44%	£2134
Difference*	–	–4977	–4978	–	–	£2469	2067	2067	–	–	–£41
Prostate cancer added	–	635,797	27,754 (2.61)	1:22	13%	£12,917	243,586	92,250 (4.23)	1:1.6	43%	£2187
Difference*	–	153,921	2866	–	–	–£2465	–567	–567	–	–	£12
MCED uptake	50%	–	–	–	–	–	122,076	46,409 (4.23)	1:1.6	22%	£2175
Difference*	–	0	0	–	–	£0	–122,077	–46,408	–	–	£0
MCED uptake	25%	–	–	–	–	–	61,038	23,204 (4.23)	1:1.6	11%	£2175
Difference*	–	0	0	–	–	£0	–183,115	–69,613	–	–	£0
Decline SOC screening^d^	–	N/A	N/A	–	–	N/A	76,346	30,945 (4.72)	1:1.5	14%	£1989
	–	N/A	N/A	–	–	N/A	–167,807	–61,872	–	–	–£186

*Difference between sensitivity analysis and primary analysis values.

^a^Only breast, cervix and colorectal cancers.

^b^Yield = cancers detected/total population of screened individuals, expressed per 1000 people.

^c^Any cancer type (including breast, cervix and colorectal missed by the established screening tests).

^d^Assumes 30% of population refuses current standard of care screening and only receives an MCED test.

^e^Calculated as (e.g.): 24,888:481,876–24,888.

^f^CDR = cancers detected/cancers expected.

Using current screening guidelines, diagnostic investigation costs are an estimated £260 million (£13 million for true positives plus £247 million for false positives), and £202 million with the incremental MCED test. But 3.7 times more cancers are detected compared to recommended screening alone. The diagnostic costs per cancer detected using the three single-cancer tests is £10,452, but only £2175 with the incremental MCED test.

### Combined screening performance and costs

**Fig. 3:**
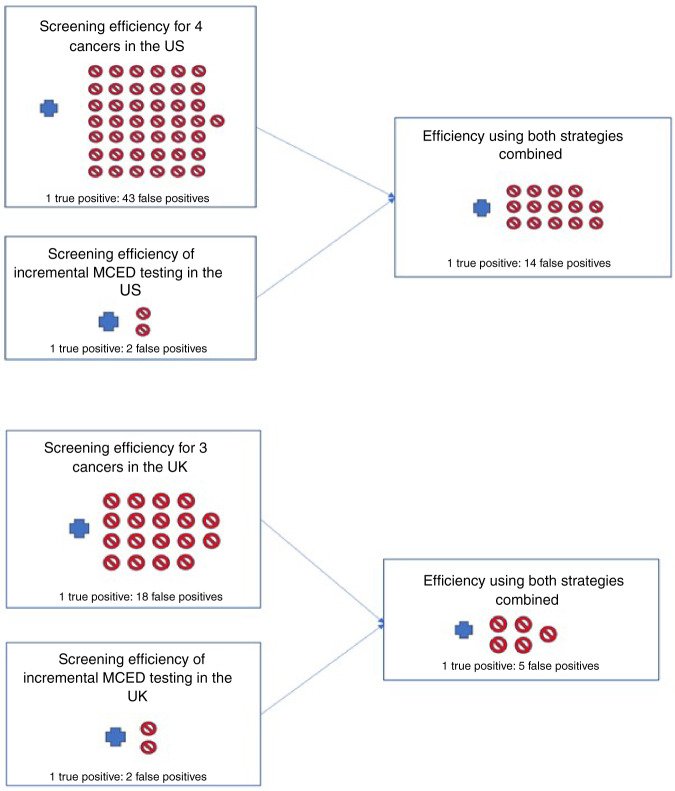
Efficiency of cancer screening under current screening paradigms and with addition of an MCED test in the United States (upper) and United Kingdom (lower). A TP:FP (true positive: false positive) of, for example, 1:43 means that for every cancer diagnosed, 43 people without cancer might undergo cancer investigations due to having a positive screening test). Both the US and UK recommend screening for breast, bowel and cervical cancers, and lung cancer screening is also recommended in the US.

Table 3 shows the outcomes based on current screening and the MCED blood test when considered together (to represent the total impact of screening in the population using both strategies), and Fig. 3 illustrates the screening efficiency. Even though the total number of screen positives is high (9,409,588 US; 726,029 UK), the total number of cancers diagnosed among them is 611,603 (US) and 117,705 UK, producing a low TP:FP of 1:14 US and 1:5 UK. The diagnostic costs per cancer ($32,461; £3,925) are still lower than those for current screening ($89,042; £10,452).

**Table 3:** Screening outcomes based on combining current screening with the incremental MCED test in the US and UK (based on Tables 1 and 2).

		US				UK			
Analysis	Variation applied for sensitivity analysis	Total positives	True positives (yield per 1000)	TP:FP ratio	Diagnostic cost per confirmed cancer diagnosis	Total positives	True positives (yield per 1000)	TP:FP ratio	Diagnostic cost per confirmed cancer diagnosis
Current screening only	–	8,247,155	189,498 (2.66)	1:43	$89,042	481,876	24,888 (3.43)	1:18	£10,452
Current screening only: using colonoscopy		5,249,001	189,498 (2.32)	1:27	$77,498				
*Current screening plus MCED testing*									
Primary analysis (100% MCED uptake)	–	9,409,588^a^	611,603^b^ (3.43)	1:14	$32,461	726,029^c^	117,705^d^ (4.05)	1:5	£3925
Sensitivity analyses:	–	–	–	–	–	–	–	–	–
Decreased cancer incidence in screening population (e.g. healthy population bias)	–20%	9,388,453	590,468 (3.31)	1:15	$33,487	723,119	114,794 (3.95)	1:5	£4005
Prostate cancer added	–	10,162,500	624,297 (3.21)	1:15	$37,265	879,383	120,004 (3.70)	1:6	£4668
Colorectal screening with colonoscopy	Colonoscopy specificity = 100%	6,411,434	611,603 (3.43)	1:9	–	–	–	–	–
Updated USPSTF lung cancer screening	22% of population eligible; 43% of lung cancers	9,705,528	612,192 (3.40)	1:15	$34,073				
MCED uptake	50%	8,828,372	400,550 (3.21)	1:21	$45,845	603,952	71,297 (4.05)	1:7	£5065
	25%	8,537,763	295,024 (3.21)	1:28	$59,718	542,914	48,092 (3.01)	1:10	£6459

^a^8,247,155 + 1,162,433 from Table 1.

^b^189,498 + 422,105 from Table 1.

^c^ 481,876 + 244,153 from Table 2.

^d^24,888 + 92,817 from Table 2.

### Sensitivity analyses

Sensitivity analyses are summarised in Tables 1–3. Assuming a 20% lower incidence of cancers, the TP:FP of both current screening and the incremental MCED blood test becomes less favourable, but remains better for an MCED test. When prostate cancer is included in the current screening paradigm, 14,312 additional true positives are identified in the US (2866 in the UK) but with no improvement to less favourable TP:FP (1:43 vs. 1:43, US; 1:22 vs. 1:18, UK). Assuming all US colorectal screening is performed with colonoscopy (where the false-positive rate is close to 0%, because it is the reference for distinguishing true positives from false positives for other screening tests), the TP:FP ratio of current screening is improved to 1:27, but this is still less favourable than the MCED test (1:1.8). Increasing the eligible population for LDCT in accordance with recent USPSTF guidance has a modest impact, mainly because the low uptake limits the number of additional screens received and cancers found. Assuming 25% uptake of the MCED blood test (Tables 1, 2) still leads to many extra cancers detected compared to current screening (105,526 US; 23,204, UK), while screening efficiency (TP:FP) and the diagnostic cost per cancer found remain the same as with 100% uptake because the reduction in the number of cancers detected and decrease in costs (using 25% instead of 100% uptake) are proportional. With 25% uptake, the combined effect of current screening and the MCED test (Table 3) yields 295,024 total detected cancers in the US (56% more than current screening alone); and 48,092 in the UK (93% more than current screening). Among people who decline current cancer screening, an MCED blood test alone could find 101,186 true cancers (148,066 false positives) in the US and 30,945 cancers (45,401 false positives) in the UK (Tables 1 and 2). Therefore, while high adherence to current standard screening is important to maximise cancer detection (for the four cancer types), an MCED blood test might be able to cover some of the gaps in recommended screening.

## Discussion

The concept of an MCED test (one test for multiple cancers) might be relatively new in public health oncology, but has been established for several decades in other disorders. Prenatal screening using the same biomarkers in maternal serum, and more recently fetal cfDNA, can detect several distinct chromosomal disorders with a single blood draw (trisomies 13, 18 and 21, albeit using a different risk estimate for each trisomy).

In recent years, there has been an increase in research on MCED tests, such that the US Food and Drug Administration held a workshop in March 2020 to discuss MCED genomic tests and how they could be evaluated and implemented [31]. Most published studies on MCED blood tests have focused on their biological characteristics, and few report screening performance [12–14]. We provide estimates of the impact in a population of the effect of current screening programmes considered together (not separately), and the first assessment of the potential impact of an MCED blood test used as part of a public health strategy alongside current screening. Randomised controlled trials (RCTs) are planned or expected, and until these report (which would take several years) our study aims to provide a preliminary examination using a simple model that is relatively easy to understand, rather than a comprehensive modelling evaluation.

Current recommended screening programmes each have established efficacy and are cost effective, with favourable TP:FP ratios. The tests are characterised by having high sensitivities (typically 70–90%), with FPRs of 5–15%. The impact of these high sensitivities is lessened by insufficient uptake of screening, and that only four cancer types are covered. Consequently, the absolute number of cancers found (2.66 per 1000 screened and CDR of 15% in the US) is modest, with a high number of diagnostic investigations among false positives for every cancer diagnosed (TP:FP, 1:43). Reported sensitivities for MCED tests tend to be lower (55% using an earlier version of the Galleri test for all cancers combined), but multiple (>50) cancers are covered and the FPR is small (0.7%), so the impact is expected to be greater. Screening efficiency is striking, with a TP:FP of only 1:1.8 using the MCED test and CDR of 34% in the US (assuming 100% uptake of the test, but 25–50% uptake is also favourable). While relative effects such as sensitivities (percentages) and relative risks for cancer mortality are important measures for single-cancer tests, equal focus should be given to absolute effects such as number of cancers found and number of cancer deaths avoided in a population when using an MCED test, because they reflect the combination of high incidence (all cancer types) and moderate test sensitivity. Individuals may also not be very compliant with all recommended screening tests, so a single test may help to address this. Our modelling used a published FPR of 0.7% [14], consistent with that seen with other tests, such as CancerSEEK (1.1%) [12]. When such tests are used in a population screening programme, quality assurance factors might increase the FPR.

We focused on the additional number of false positives and cancers found when adding an MCED test to current screening. Even when looking at the total impact in a population of current and MCED screening together (Fig. 3), the TP:FP was more favourable than current screening alone (1:14 and 1:5 for the US and UK, respectively, compared to 1:43 US and 1:18 UK) because many more cancers are found using an MCED test. These estimates are based on the relatively low uptake to lung cancer screening programmes in the US, and programmes may start in the UK in the near future. If uptake increases, this would improve the outcomes associated with current recommended screening (Tables 1–2), but outcomes using an MCED test should only be modestly reduced because lung cancer represents less than 10% of all cancers detectable by the MCED test we used in our analyses. Also, the revised eligibility criteria from the USPSTF only covers 40% of all lung cancers.

In the UK, several thousand lives are saved each year through current cancer screening (e.g. 1700 breast and 2400 bowel cancers) [24]. About 18% of all cancers are diagnosed at stage IV but they represent 45% of all cancer deaths [32]. Shifting stage at diagnosis from IV to I–III is estimated to reduce the cancer death rate by 15–24% [32]. If such stage shifts can be achieved in practice, an MCED test that can detect 211,052 (US) or 46,409 (UK) additional cancers with 50% uptake, could save several thousand extra lives annually but this needs to be demonstrated in prospective studies.

Diagnostic investigations for cancer are expensive, with psychological morbidity for patients and families [33]. There is a substantial difference in the cost of diagnostic investigations per cancer detected between current screening ($89,042 or £10,452) and application of an MCED test ($7060 or £2175) even with the extreme assumption of 100% uptake, and that all false positives have further investigations. A major requirement of an effective MCED test is that it can identify the tumour of origin to specific tissues and anatomic sites. Otherwise, diagnostic investigations could be unfocussed leading to unnecessary or inappropriate imaging (with more total-body radiation exposure), higher costs and a longer time to definitive diagnosis to locate the primary tumour. This creates anxiety for the patient and frustration for the clinician.

Large RCTs with long follow-up were used to evaluate previous cancer screening tests, but innovative and complementary approaches to evidence generation are needed for rapidly evolving MCED genomic tests that detect large numbers of cancers and can demonstrate stage shift and cancer-specific mortality benefits in a shorter timeframe. This is expected to be a combination of RCTs and real-world evidence prospective longitudinal studies. Box 1 displays key features of a successful MCED test.

An effective MCED test, using a single blood draw, should be appealing and convenient to people, including those of lower socioeconomic status and other hard to reach groups with lower uptake of current screening. Population cancer screening is expensive in high-income countries, and this will become even more pertinent in middle income countries where ageing populations lead to higher cancer rates, with issues over affordability of expensive drugs to treat cancer when diagnosed late, as well as access to current screening. One important consideration is whether having an MCED test deters people from participating in current screening programmes whose tests may have higher sensitivities. However, in the CancerSEEK study MCED results were reported and acted upon, but participants continued to have high adherence to standard screening [12].

Overdiagnosis is a known harm of screening, where imaging, direct tissue visualisation and protein biomarkers have limited or no ability to discriminate indolent (precancer or cancer that never progresses or cause symptoms) from invasive cancers [34]. However, genomic testing utilised in an MCED blood test is focused on circulating tumour DNA and leverages the biological mechanism of cancer, potentially minimising overdiagnosis [35]. Ongoing prospective studies of various MCED tests will provide more information on overdiagnosis.

We focused on how an MCED blood test could complement current screening, in which people who are not diagnosed with any of the four cancer types (via standard of care screening) or those ineligible for the recommended tests receive the MCED test independently. Other approaches could be based on current screening and an MCED blood test performed at the same time to give a single result among those eligible for recommended screening, and/or the MCED test is only offered to people who are ineligible for recommended screening. The effect on screening performance when combining tests requires knowing the extent to which current and MCED tests are independent (i.e. whether they largely identify the same people with cancer or they detect different people). Ongoing large-scale studies will determine this [36, 37]. In the CancerSEEK study, people diagnosed with cancer who were MCED test positive did not overlap with screen positives using standard screening, indicating potentially independent effects [12]. Screening performance could be more efficient (higher sensitivity and/or lower FPRs) if combining an MCED test with standard screening to produce a single test result; also avoiding potential issues over having two screening test results given separately. Future analyses could examine the value and cost effectiveness of MCED screening in people younger than that recommended for current screening (e.g. <45 years), particularly if they have high-risk characteristics.

Our study had limitations. First, we did not estimate reductions in cancer mortality or advanced cancers diagnosed (stage shift) because this information is not yet available for any MCED test; and reductions in cancer deaths would be influenced by the sensitivity of the MCED test among early stage cancers. However, the outcomes we included (number of screen positives and cancers detected, and diagnostic costs) are clinically relevant and would be part of a fuller assessment of MCED tests. Second, our analyses did not consider precancerous lesions for cervical cancer and precancerous polyps for colorectal cancer so there may be additional benefits to current screening that are not quantified here (the TP:FP ratio would be more favourable if precancerous features were considered true positives). Third, although we used published sources, different estimates of sensitivity, FPR, uptake and cancer incidence could yield different outcomes, as well as allowance for interval cancers and uptake of diagnostic testing following a screen-positive result. We used SEER cancer incidence, which tends to have more racial diversity and greater economic disadvantage than is found in areas without SEER registries [38]. Incidence will depend on which individuals have the MCED test (e.g. they could have healthier lifestyles), and also overdiagnosis (100% uptake of the test probably yields a higher overall incidence than the general population due to the detection of indolent cancers not found in the absence of screening). Furthermore, our estimates of sensitivity and FPR for the MCED test come from a study of symptomatic people undergoing cancer investigations (the same with nearly all other MCED studies), which represents the best evidence to date. Although we used stage-specific sensitivities for each cancer type, the sensitivity of MCED tests for lower stage cancers (the main target of screening) may or may not be substantially different in asymptomatic people. It also needs to be determined whether MCED test performance among people who are screen negative for current recommended screening is the same as in those who have had no screening at all (which we assumed), and MCED test performance also needs to be ascertained in people who decline current screening. Fourth, we used diagnostic costs based on commercial payers that may not be generalisable within certain US and UK populations. However, our analyses used the same diagnostic cost estimates for recommended and MCED screening. Fifth, for simplicity we only provided the effect of screening in a single year (essentially a snapshot), acknowledging that MCED tests would be evaluated over several years, in which individuals have multiple screens. Once data are available on the ideal frequency of screening per person and also uptake at successive screens, future modelling can incorporate these, with consideration of annualised costs and discounting. Finally, MCED tests need to be able to identify the location of the primary tumour to guide further workup, and we did not allow for the cost of incorrect localisation. For the MCED test we considered, the localisation appears to be correct 93% of the time when a cancer signal is detected [14], and thus increases in diagnostic workup costs following incorrect localisation would be a small increment to the overall costs. Ongoing studies can further estimate the level of accuracy and according to cancer stage.

No MCED test is licensed for use so none have a price yet (and as with many therapeutic drugs would be determined after definitive RCTs have completed and negotiation with payers). The cost of an MCED test will depend on where blood samples are taken. Current cancer screening (except for bowel using FIT) is undertaken at specialist screening units. However, screening for cardiovascular disease involving a blood draw for lipid levels is already performed in primary care. Taking blood samples for an MCED test in the primary care or community settings, or perhaps local pharmacies, might therefore be possible without substantial extra costs, especially because no special on-site processing is required. Future cost-effectiveness analyses (including cost per life year gained) will incorporate the cost of the tests, diagnostic investigations and cancer treatments, as well as the costs of setting up and maintaining the screening programme. The potential shift in the stage at diagnosis by using an MCED test will also matter because treatment costs for earlier stage cancer (many requiring surgery only) are usually lower than that of cancers detected at later stages (requiring systemic, often expensive, therapies) [39].

Current screening programmes are each highly effective, while the application of MCED tests has the potential to significantly improve upon this because they can detect many more cancer types and number of cancer cases with a low FPR. Our analyses do not examine outcomes and costs of MCED testing as a replacement for current screening, but rather what the possible effect might be of using MCED testing alongside current screening. MCED blood tests could allow public health policy to move away from screening for individual cancer types only to include screening individuals for multiple cancers. As evidenced by the increased CDR from our analysis, adding an MCED test to guideline-recommended screening could efficiently identify deadly cancer types that would not be found with any current screening programme while minimising additional false-positive results. MCED tests should not be used in routine practice without clear evidence on efficacy, harms and other performance measures. Our findings should stimulate further research on the effectiveness and health economic assessment of MCED tests, using additional measures of screening impacts as we present.

Box 1 Proposed criterion for a successful multi-cancer early detection test

- ✓  Sufficiently high sensitivity with a high cancer detection rate (absolute number of cancers detected), and reduction in cancer deaths.
- ✓ Minimise patient harm through a fixed, very low false-positive rate (<1%), resulting in a high positive predictive value
- ✓ Able to identify most types of cancer, and the majority of deadly cancers
- ✓ Potential to minimise/avoid overdiagnosis by being more sensitive to and preferentially detecting more lethal cancers
- ✓  Able to accurately localise cancer to specific organs in order to efficiently direct diagnostic workup
- ✓Simple test to use, convenient for people to access, and no specialist equipment of staff required to administer the test: aim is to maximise uptake and maintain adherence over several years (screens)
- ✓ Supported by robust analytical and clinical validation at population scale

## Supplementary information

Supplementary information

Reproducibility checklist

## Data Availability

All the parameters used in the modelling are available from the publications cited. Further details of the modelling and parameters can be obtained by request from the authors.
