# Supplementary material for: Estimating the population health impact of a multi-cancer early detection genomic blood test to complement existing screening in the US and UK
Source: Br J Cancer. 2021 Aug 23;125(10):1432–42. doi: 10.1038/s41416-021-01498-4 (PMC8575970; doi:10.1038/s41416-021-01498-4)
Supplement: Supplementary file 1 — Supplementary information [file 41416_2021_1498_MOESM1_ESM.docx]

**Supplementary Data**

**Estimating the Population Health Impact of a Multi-cancer Early Detection Genomic Blood Test to
Complement Existing Screening in the US and UK**

**Supplemental Table 1.** Inputs for US and UK estimates using current screening guidelines

|  |  | **United States (USPSTF)** | | | | | |
| --- | --- | --- | --- | --- | --- | --- | --- |
|  | **Modality** | **% adults age 50-79 eligible for current recommended screening** | **Adherence** | | **Screening Interval** | **Sensitivity** | **Specificity (1-false-positive rate)** |
| Breast | Mammography | 90% of women | 77·6% | | Annually^5^ | 87% | 89% |
| Colorectal | Cologuard | 93% | 67·7% | | Every 3 years | 92% | 87% |
| Cervical | Cotesting^1^ | 63% of women | 95% | | Every 5 years | 95% | 85% |
| Lung | LDCT^2^ | 7%^3^ | 14·4% | | Annually | 84·9% | 87·2% |
|  |  | **United Kingdom (National Screening)** | | | | | |
| Breast | Mammography | 77% of women | 70·5% | Every 3 years | | 87% | 97.2% |
| Colorectal | FIT^4^ | 68% | 57·7% | Every 2 years | | 79% | 94% |
| Cervical | Cotesting^1^ | 60% of women | 76·2% | Every 5 years | | 95% | 85% |

USPSTF = United States Preventive Services Task Force;

^1^ Cotesting = Cervical cytology and hrHPV testing together. There is uncertainty in the estimate of uptake of cervical cancer screening as the BRFSS survey focuses on PAP testing rather than cotesting (e.g. women may not be aware of whether they had cotesting or not).

^2^ LDCT = Low-dose computed tomography

^3^ Representing 27% of lung cancers as LDCT is based on risk, not age hence many smokers and ex-smokers are not currently eligible for LDCT screening.

^4^ FIT = Faecal immunochemical test

^5^ USPSTF recommends biennial screening but we use the American Cancer Society and NCCN recommendations of annual screening (for some age groups) in order to maximise the benefits associated with current screening. Oeffinger et al, JAMA 2015;314(15): 1599-1614 and <https://www.nccn.org/professionals/physician_gls/pdf/breast-screening.pdf>

**Supplemental Table 2.** Sensitivities of an MCED test by cancer site and stage (detected cancer/total cancer)^

| **Cancer Site/Type** | **Stage I** | **Stage II** | **Stage III** | **Stage IV** |
| --- | --- | --- | --- | --- |
| Anus | 33·3% (1/3) | 60·0% (3/5) | 100·0% (5/5) | * |
| Bladder | 60·0% (3/5) | 66·7% (2/3) | 50·0% (1/2) | 0·0% (0/1) |
| Breast | 4·9% (5/102) | 41·8% (46/110) | 88·9% (24/27) | 100·0% (8/8) |
| Cervix | 25·0% (2/8) | 100·0% (1/1) | 100·0% (2/2) | * |
| Colon/Rectum | 50·0% (7/14) | 72·7% (16/22) | 75·6% (31/41) | 93·3% (42/45) |
| Esophagus | 16·7% (1/6) | 75·0% (6/8) | 94·1% (16/17) | 100·0% (19/19) |
| Gallbladder | 0·0% (0/3) | 100·0% (1/1) | 66·7% (2/3) | 100·0% (4/4) |
| Head and Neck | 85·7% (6/7) | 92·3% (12/13) | 93·8% (15/16) | 92·3% (24/26) |
| Kidney | 0·0% (0/37) | 25·0% (1/4) | 50·0% (2/4) | 81·8% (9/11) |
| Liver/Bile-duct | 60·0% (3/5) | 85·7% (6/7) | 85·7% (6/7) | 100·0% (10/10) |
| Lung | 18·6% (11/59) | 78·3% (18/23) | 87·5% (63/72) | 91·5% (97/106) |
| Lymphoma | 20·0% (3/15) | 75·0% (21/28) | 77·8% (21/27) | 79·5% (31/39) |
| Melanoma | 0·0% (0/3) | 0·0% (0/1) | * | 100·0% (3/3) |
| Ovary | 50·0% (2/4) | 0·0 % (0/2) | 92·0% (23/25) | 100·0% (6/6) |
| Pancreas | 66·7% (8/12) | 78·6% (11/14) | 87·5% (14/16) | 95·2% (40/42) |
| Plasma Cell Neoplasm | 50·0% (5/10) | 69·2% (9/13) | 100·0% (11/11) | * |
| Prostate | 0·0% (0/39) | 4·4% (5/113) | 10·5% (2/19) | 76·5% (13/17) |
| Sarcoma | 100·0% (2/2) | 0·0% (0/4) | 40·0% (2/5) | 66·7% (4/6) |
| Stomach | 33·3% (1/3) | 100·0% (4/4) | 100·0% (2/2) | 100·0% (8/8) |
| Thyroid | 0·0% (0/2) | 0·0% (0/1) | * | 0·0% (0/1) |
| Urothelial Tract | 100·0% (1/1) | 50·0% (2/4) | * | * |
| Uterus | 21·9% (16/73) | 66·7% (2/3) | 60·0% (3/5) | 66·7% (2/3) |

Notes: Cancer site/type grouped for reporting purposes and reflect more than 50 cancer types (1). Additional cancer sensitivities included in calculations but not staged: Lymphoid Leukemia 57.9%, and Myeloid Neoplasm 0.0%. Cancers denoted as “Other” by Liu et al were not included in calculations (2).

^ Non-decreasing sensitivity by stage from isotonic regression was used in the analysis, due to limited sample size, no material effect on projected outcomes.

*Some sensitivity values were not available due to small sample sizes.

**Supplemental Table 3a (US) and 3b (UK).** Summary of diagnostic work-up cost for US (3a) and UK (3b) following a positive screening test result

**3a.** US

| **Cancer** | **Procedure*** | **Code** | **Additional Procedures** | **Code** | **Total** |
| --- | --- | --- | --- | --- | --- |
| Lung and Bronchus | LDCT for lung ca screen | G0297 | Biopsy, lung or mediastinum, percutaneous needle | 32405 | $3,730·99 |
| Colon and Rectum | Colonoscopy, flexible; diagnostic, including collection of specimen(s) by brushing or washing, when performed (separate procedure) | 45378 | NA | ·· | $799·78 |
| Lymphoma | CT Chest w/ Contrast | 71260 | Biopsy or excision of lymph node(s), by needle, superficial | 38505 | $3,593·96 |
| Breast Cancer | Mammogram Bilateral | 77066 | Biopsy of breast, percutaneous, needle core, not using imaging guidance | 19100 | $3,569·97 |
| Pancreas | CT Abd W W/O Contrast | 74170 | Transendoscopic ultrasound-guided intramural or transmural fine-needle aspiration/biopsy(s) Esophagogastroduodenoscopy, flexible, transoral | 43242 | $4,019·00 |
| Head and Neck | E&M Moderate to High Severity (40 minutes face-to-face) | 99215 | Diagnostic laryngoscopy | 31575 | $719·10 |
| Liver and Intrahepatic Bile Duct | US Abdomen Complete | 76700 | Biopsy of liver, needle, percutaneous | 47000 | $3,462·88 |
| Ovary | Ca screen;pelvic/breast exam | G0101 | US Abdomen Complete | 76700 | $399·69 |
| Stomach | Esophagogastroduodenoscopy, flexible, transoral; diagnostic, including collection of specimen(s) by brushing or washing, when performed | 43235 | NA | ·· | $274·37 |
| Esophagus | Esophagogastroduodenoscopy, flexible, transoral; diagnostic, including collection of specimen(s) by brushing or washing, when performed | 43235 | NA | ·· | $274·37 |
| Kidney | CT Abd & Pelvis W W/O Contrast | 74178 | Renal biopsy; percutaneous, by trocar or needle | 50200 | $4,053·11 |
| Prostate | MRI Pelvis W W/O Contrast | 72197 | Biopsy, prostate; needle or punch, single or multiple, any approach | 55700 | $4,970·69 |
| Multiple Myeloma | Comprehensive (13 Tests - Multiple Codes) | Multiple Codes | NA | ·· | $158·93 |
| Leukemia | Bl smear w/diff wbc count | 85007 | NA | ·· | $26·61 |
| Indeterminate | CT Extremity Upper W/ Contrast | 73201 | NA | ·· | $896·13 |
| Uterine | Ca screen;pelvic/breast exam | G0101 | US Abdomen Complete | 76700 | $399·69 |
| Bladder | CT Abd & Pelvis W W/O Contrast | 74178 | Renal biopsy; percutaneous, by trocar or needle | 50200 | $4,053·11 |
| Melanoma | Punch Skin Biopsy Single Lesion | 11104 | NA | ·· | $314·20 |
| Cervical | Ca screen;pelvic/breast exam | G0101 | NA | ·· | $111·67 |
| Anal | Colonoscopy, flexible; diagnostic, including collection of specimen(s) by brushing or washing, when performed (separate procedure) | 45378 | NA | ·· | $799·78 |

*Note: all workups assumed to begin with a CBC (HCPCS code 85025); commercial costs assumed to be 2.3x Medicare rate.

**3b.** UK

| **Cancer** | **Initial visit** | **Code** | **Procedure** | **Code** | **Additional Procedures** | **Code** | **Total cost** |
| --- | --- | --- | --- | --- | --- | --- | --- |
| Lung and Bronchus | Respiratory Medicine | 340 | CT of One Area, without Contrast, 19 years and over | RD20A | Biopsy of Lung | YD03Z | £1,171·00 |
| Colon and Rectum | Gastroenterology | 301 | Diagnostic Colonoscopy, 19 years and over | FE32Z | ·· | ·· | £667·00 |
| Lymphoma | Clinical Haematology | 303 | Computerised Tomography Scan of Three Areas, with Contrast | RD26Z | Core Needle Biopsy of Axillary Lymph Nodes | YJ04Z | £692·00 |
| Breast Cancer | Gynaecology | 502 | Diagnostic Imaging | RD21A | Bilateral Core Needle Biopsy of Lesions of Breasts | YJ01Z | £540·00 |
| Pancreas | Hepatology | 306 | Computerised Tomography Scan of Three Areas, with Contrast | RD26Z | Endoscopic Ultrasound Examination, of Hepatobiliary or Pancreatic Duct, with Biopsy or Cytology | GB12Z | £1,006·00 |
| Head and Neck | ENT | 120 | Medical Oncology | 370 | Diagnostic Nasopharyngoscopy, 19 years and over | CA71A | £537·00 |
| Liver and Intrahepatic Bile Duct | Hepatology | 306 | Ultrasound Scan with duration of less than 20 minutes, without contrast | RD40Z | Percutaneous Punch Biopsy of Lesion of Liver, 19 years and over | YG11A | £1,063·00 |
| Ovary | Gynaecology | 502 | Gynaecological Oncology | 503 | Ultrasound Scan with duration of less than 20 minutes, without contrast | RD40Z | £348·00 |
| Stomach | Gastroenterology | 301 | Diagnostic Endoscopic Upper Gastrointestinal Tract Procedures, 19 years and over | FE22Z | NA | ·· | £555·00 |
| Esophagus | Medical Oncology | 370 | Diagnostic Endoscopic Upper Gastrointestinal Tract Procedures, 19 years and over | FE22Z | NA | ·· | £624·00 |
| Kidney | Urology | 101 | Computerised Tomography Scan of Three Areas, with Contrast | RD26Z | Percutaneous Needle Biopsy of Lesion of Kidney, 19 years and over | YL20A | £980·00 |
| Prostate | Urology | 101 | Magnetic Resonance Imaging Scan of One Area, with Pre- and Post-Contrast | RD03Z | Transrectal Ultrasound Guided Biopsy of Prostate | LB76Z | £639·00 |
| Multiple Myeloma | Clinical Haematology | 303 | Medical Oncology | 370 | NA | ·· | £554·00 |
| Leukemia | Clinical Haematology | 303 | NA | ·· | NA | ·· | £279·00 |
| Indeterminate | Medical Oncology | 370 | Computerised Tomography Scan of Three Areas, with Contrast | RD26Z | NA | ·· | £374·00 |
| Uterine | Gynaecology | 502 | Gynaecological Oncology | 503 | Ultrasound Scan with duration of less than 20 minutes, without contrast | RD40Z | £348·00 |
| Bladder | Urology | 101 | Computerised Tomography Scan of Three Areas, with Contrast | RD26Z | Percutaneous Needle Biopsy of Lesion of Kidney, 19 years and over | YL20A | £980·00 |
| Melanoma | Dermatology | 330 | Excision or Biopsy, of Lesion of External Nose | CA16Z | NA | ·· | £276·00 |
| Cervical | Gynaecology | 502 | Gynaecological Oncology | 503 | NA | ·· | £308·00 |
| Anal | Gastroenterology | 301 | Diagnostic Colonoscopy, 19 years and over | FE32Z | Diagnostic Flexible Sigmoidoscopy, 19 years and over | FE35Z | £978·00 |

**Supplemental Figure 1.** New cancer cases and cancer deaths for adults age 50-79, by site, estimated for 2020, United States


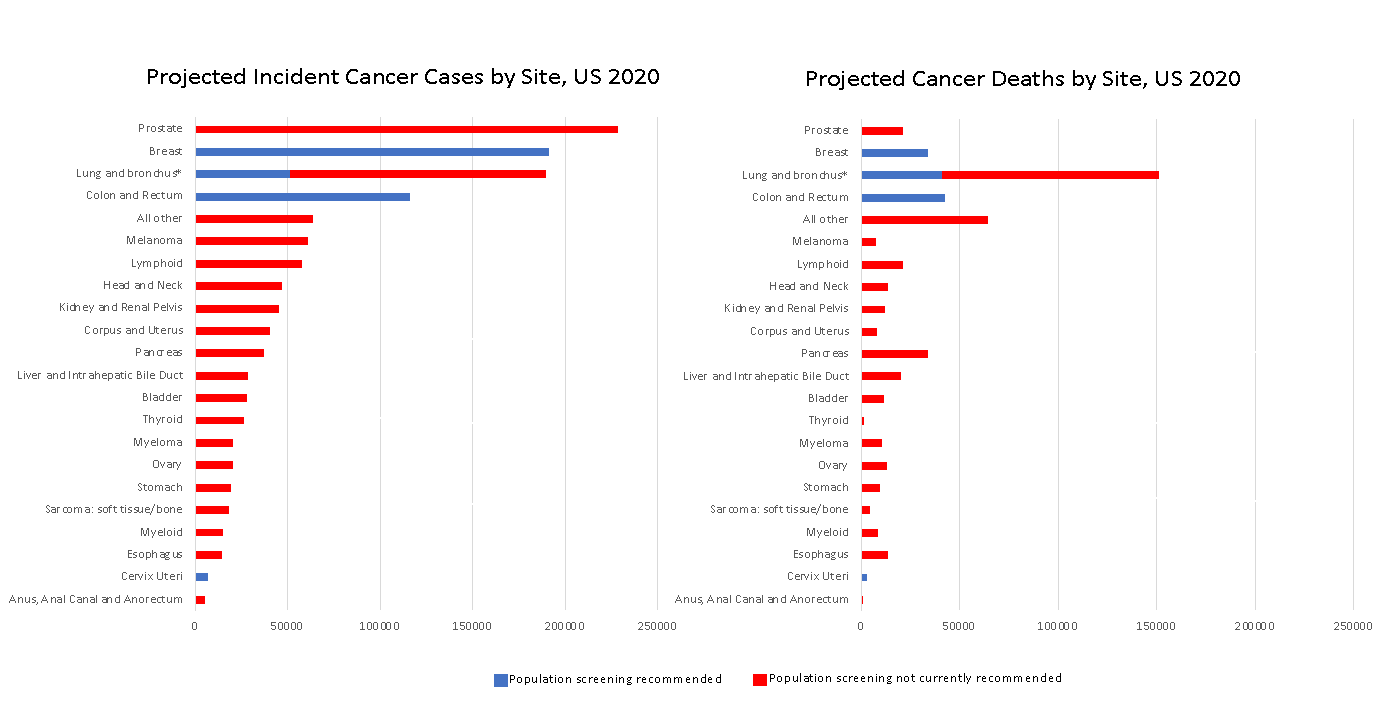


* For lung cancer, only those considered at highest risk are included in the current screening recommendations.

Blue represents current population screening recommended cancers with USPSTF Grade A or B recommendations. For cancer incidence, the blue bars together indicate that 29% of all cancers are among the four types for which screening is currently recommended. For cancer deaths, the blue bars together indicate that 24% of all deaths are among the four types for which screening is currently recommended.

**Source:** Underlying data obtained from SEER/Stat incidence rates for ages 50-79 for years 2000-2016. Projections based on US population estimate of 107,000,000 for this age range in 2020.

**Supplemental Text 1**

**Methods**

The potential impact of a multi-cancer early detection (MCED) blood test when used alongside current screening was modelled in the United States (US) and United Kingdom (UK), focusing on incident cancers. The target population was all adults aged 50-79 years in the US and 45-74 years in the UK: 107,000,000 (estimated) and 21,834,470, respectively for 2020. The UK age range was different only because incidence figures in the UK are reported in these age bands: 45-54, 55-64, and 65-74. The diagram below illustrates the components used for the modelling and the outcomes produced:


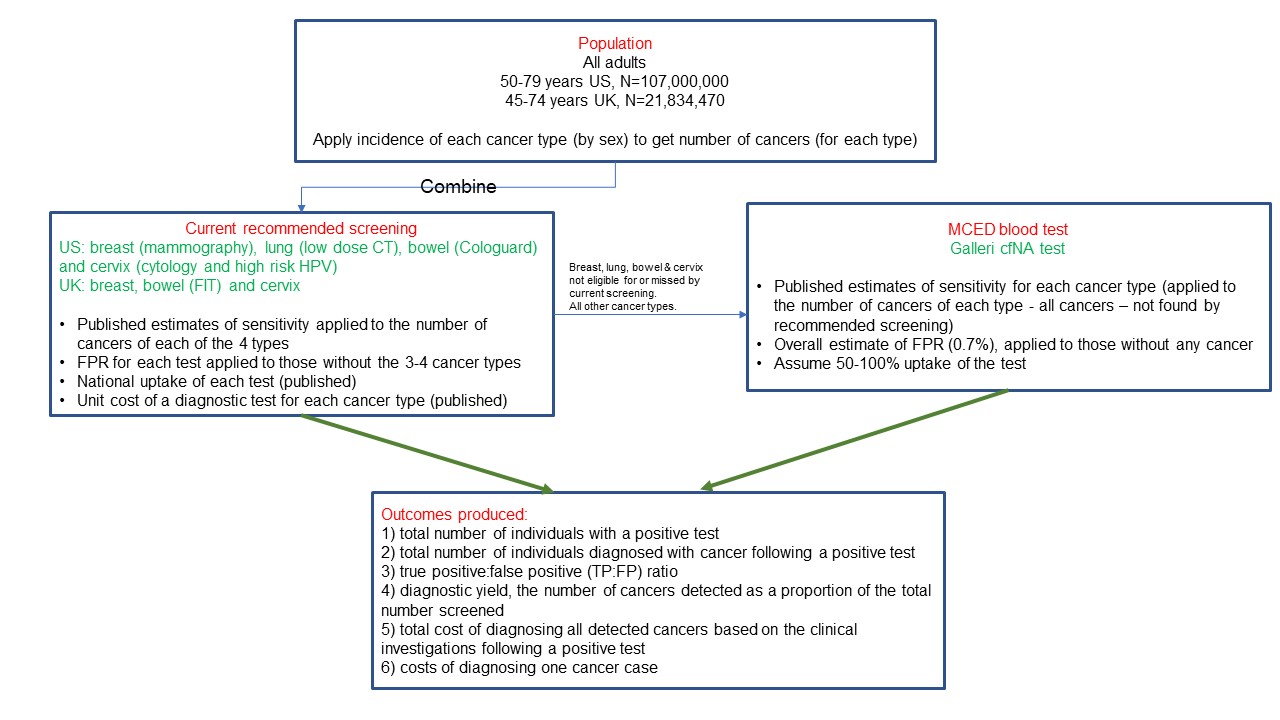


*Incidence*

Cancer incidence rates for the US were obtained from SEER for adults aged 50-79 years in 2016.^1^ UK incidence rates came from the Office for National Statistics in the UK^2^ supplemented with the proportion of ER-/PR-positive breast cancer cases and the distribution of lymphoma incidence by stage from US data,^1^ as they were unavailable for the UK.

*Current guideline-recommended screening parameters*

Key model parameters are shown in **Supplemental Table 1**. Using United States Preventive Services Task Force (USPSTF) guidelines, we included breast cancer screening using mammography for women aged 50-79 years; lung cancer screening using low-dose computed tomography (LDCT) for adults aged 55-80 years who have a 30 pack-year smoking history and currently smoke or have quit within the past 15 years; cervical cancer screening using cytology and high-risk human papillomavirus (hrHPV) testing for women aged 50-65; and colorectal cancer screening using a fecal DNA test (Cologuard) for adults aged 50-75 years.^3-7^ Nationally representative estimates of uptake came from the Behavioral Risk Factor Surveillance System (BRFSS).^8^ New guidance around lung cancer screening eligibility is currently under consideration by the USPSTF but unlikely to be adopted in routine practice immediately, therefore our models used current recommendations.

In the UK, breast and cervical cancer screening modalities are the same as in the US, while a common modality for colorectal screening in the UK is the faecal immunochemical test (FIT). Uptake of screening tests in the UK were obtained from a recent report.^9^ Screening intervals were obtained from UK-specific guidance.^10^

*Screening performance for currently recommended screening and MCED*

Screening performance (sensitivity and false positive rate [FPR]) for each of the four currently recommended tests were as published (6–9) [sensitivity: proportion of people with cancer who have a positive test; FPR: proportion of people without cancer who have a positive test]. The Galleri blood test utilizes targeted methylation analysis of circulating cell-free DNA (cfDNA) to detect multiple cancer types, and sensitivities by cancer type are available.^11^ We assumed 100% uptake for an MCED blood test (to reflect possible maximum gains and maximum diagnostic costs); and test sensitivity that varies by cancer type and stage (**Supplemental Table 2**; overall sensitivity 55%); and FPR of 0.7% (2).^11^ An isotonic regression, using the pool adjacent violators algorithm in the software R, was applied to ensure sensitivity was non-decreasing with increasing stage within a specific cancer type. It is important to note that the performance results for MCED are from a case-control study and thus may not represent the true effectiveness of MCED in clinical practice, while those for single cancer screening are from clinical practice or randomized clinical trials.

All calculations are for incident screens – i.e. reflect a typical year within an ongoing screening program. In the first year of a screening program, cancer incidence increases relative to the absence of screening, as cancers that would be incident in future years are “pulled forward” into the current year. The number of cancers detected in a prevalent screen, however, is dependent on the rate at which undetected cancers progress and has substantial uncertainty. Making a simplifying assumption of no overdiagnosis, the total incidence of cancer in subsequent years of an ongoing screening program is equal to the total incidence in the absence of that program, as cancers pulled forward from the current year to earlier years are balanced by cancers pulled from future years to the current year. Notably, because of this balance, the number of cancers detected by screening in an incident screen is unaffected by lead time bias, though the stage at diagnosis and the clinical benefit of diagnosing a cancer early will be impacted. Screening will result in some overdiagnosis, however, and those overdiagnosed cancers could (in future analyses) be added to the annual incidence to estimate a total incidence with screening.

The number of TP for a single cancer screening was calculated as:

uptake × sensitivity × overall incidence

where uptake is the number of adults in the target population multiplied by the percentage adherent to guidelines for that screening test, sensitivity is the sensitivity of the test for invasive cancer (i.e., not including precancerous lesions), and overall incidence is the incidence across all stages of the cancer being screened for.

The number of true-positives (TPs, i.e., cancers) and false-positives (FPs) in the target age group (50-79 in the US, and 45-74 in the UK) identified by the recommended screening tests for a one-year period were calculated by applying the proportion of cancers potentially screened (100% for breast [i.e., all breast cancers in women aged 50-79 years meet the eligibility criteria for recommended screening], cervix and colorectal cancer, and 27% for lung cancer [i.e., only 27% of people with lung cancer aged 50-79 meet the eligibility criteria for current screening]), the screening interval, uptake, sensitivity, and FPR to cancer incidence rates for the four cancer types in the US (**Supplemental Table 1**). Lung cancer was excluded from the UK estimates because screening is not currently recommended there. Although a particular individual could be screened within two or more different screening programs in practice, we treated each screening test independently so that all potential diagnostic costs are captured.

Because we are focused on the effect of MCED that is incremental to single cancer screening, cancers detected by single cancer screening are excluded from the pool of cancers potentially detected by MCED. Thus, for a single cancer type, the number of TP for the MCED test was calculated as:

MCED uptake × Σ_stages_ [MCED sensitivity_stage_ × (incidence_stage_-cancers detected by single cancer screening)]

This calculation is a sum over invasive cancer stages (stage I – IV), where MCED uptake is the number of adults in the target population multiplied by an assumed adherence rate, MCED sensitivity_stage_ is the sensitivity of the MCED test by stage, incidence_stage_ is the incidence of that cancer type by stage, and cancers detected by single cancer screening is computed as above for cancers with recommended screenings and 0 (zero) for those without. The total number of cancers detected by MCED is then calculated by summing detected cancers in each cancer type. This calculation estimates the number of cases the MCED would detect by the stage at which the cancers clinically present, but does not calculate the stage at which they are detected by MCED. Because it does not predict stage at detection, this calculation relies only on the observed stage distribution of incident cancers.

These calculations embed several assumptions. The calculations assume that are no interval cancers with current single cancer screenings, and thus that all observed cancers are available for detection. This assumption creates a slight bias to increase the number of cancers detected by single cancer screenings, which in turn reduces the number of cancers that are potentially detected by MCED.

These calculations also include the assumption that single cancer screenings detect the same fraction of observed incident cancers in all stages. In practice, current screenings likely represent a larger fraction of early stage cancer diagnoses that late stage diagnoses among those cancers with available screenings. This simplifying assumption results in relatively more early stage cancers available for detection by MCED and relatively fewer late stage cancers. Because the MCED sensitivity increases with stage, this assumption again creates a modest bias to reduce the number of cancers detected by MCED.

As noted above, these calculations assume no overdiagnosis. This assumption reduces the number of cancers detected by MCED by accounting for only cancers that would have been incident in the absence of MCED. The harms associated with overdiagnosed cancers, however, should be accounted for separately and are discussed in the limitations to this analysis.

The number of FPs for each screening test was computed as:

((uptake – individuals with cancer detected) × false-positive rate) / screening interval

where individuals with cancer detected includes only those within the fraction adherent to screening; and screening interval is, for example, 1 for annual screening and 3 for screening every 3 years.

The analyses produced the number of additional cancers (all cancer types) that could be detected by an MCED test during a one-year period, separate to those found through recommended screening. Additional cancers found by an MCED blood test includes breast, cervix, colorectal and lung cancers among people aged 50-79 years who are not eligible for current screening based on existing criteria, as well as those among eligible people who were screened but were missed by the test. The additional number of FPs was determined by applying the 0·7% rate to the target population size (minus the number of cancers detected by recommended screening).

*The two scenarios*

The target population was all adults aged 50-79 years in the US and 45-75 years in the UK. Outcomes were estimated for two scenarios, outlined in **Figure 1** (for the US estimates). The first scenario assumes that eligible individuals are screened using recommended tests only within the specified age range for these tests. The second scenario applies an MCED blood test to anyone not diagnosed with cancer following a positive screen from any of the currently recommended tests (i.e., those who were screen-negative and those who were screen-positive but without the cancer being screened for), and also to all adults in the target age group who were ineligible for recommended screening. Therefore, the second scenario represents any type of cancer found in addition to those identified through current screening paradigms, with a small proportion who would be expected to be test-positive under both standard screening and with an MCED test.

**References**

1. Surveillance Epidemiology and End Results (SEER) Program. SEER*Stat Database: Incidence - SEER 18 Regs Research Data + Hurricane Katrina Impacted Louisiana Cases, Nov 2018 Sub (1975-2016 varying) - Linked To County Attributes - Total U.S., 1969-2017 Counties. In: National Cancer Institute D, Surveillance Research Program, released April 2019, based on the November 2018 submission. 2019th ed.

2. Cancer survival in England - adults diagnosed - Office for National Statistics [Internet]. [cited 2019 Jul 29]. https://www.ons.gov.uk/peoplepopulationandcommunity/healthandsocialcare/conditionsanddiseases/datasets/cancersurvivalratescancersurvivalinenglandadultsdiagnosed

3. United States Preventive Services Task Force. USPSTF A and B Recommendations [Internet]. 2019. https://www.uspreventiveservicestaskforce.org/Page/Name/uspstf-a-and-b-recommendations/

4. Imperiale TF, Ransohoff DF, Itzkowitz SH, Levin TR, Lavin P, Lidgard GP, et al. Multitarget Stool DNA Testing for Colorectal-Cancer Screening. N Engl J Med. 2014 Apr 3;370(14):1287–97.

5. Kim JJ, Burger EA, Regan C, Sy S. Screening for Cervical Cancer in Primary Care: A Decision Analysis for the US Preventive Services Task Force. JAMA. 2018 Aug 21;320(7):706.

6. Lehman CD, Arao RF, Sprague BL, Lee JM, Buist DSM, Kerlikowske K, et al. National performance benchmarks for modern screening digital mammography: update from the Breast Cancer Surveillance Consortium. Radiology. 2017 Apr;283(1):49–58.

7. Pinsky PF, Gierada DS, Black W, Munden R, Nath H, Aberle D, et al. Performance of Lung-RADS in the National Lung Screening Trial: A Retrospective Assessment. Ann Intern Med. 2015 Apr 7;162(7):485–91.

8. Centers for Disease Control and Prevention. Prevalence & Trends Data. Centers for Disease Control and Prevention, National Center for Chronic Disease Prevention and Health Promotion, Division of Population Health [Internet]. 2015. Available from: https://www.cdc.gov/brfss/brfssprevalence/

9. Report of the Independent Review of Adult Screening Programmes in England [Internet]. 2019. Report No.: 01089. Available from: https://www.england.nhs.uk/wp-content/uploads/2019/02/report-of-the-independent-review-of-adult-screening-programme-in-england.pdf

10. UK NHSN. NHS Screening. <https://www.nhs.uk/conditions/nhs-screening/>. Updated 2020.

11. Liu MC, Oxnard GR, Klein EA, Swanton C, Seiden MV, Cummings SR, et al. Sensitive and specific multi-cancer detection and localization using methylation signatures in cell-free DNA. Annals of Oncology. 2020;31(6):745–59.
